# Severe Uncontrolled Pancreatogenic Diabetes in a Patient With Prior Alcoholic Pancreatitis and Partial Pancreatectomy

**DOI:** 10.7759/cureus.113222

**Published:** 2026-07-23

**Authors:** Reza Alavi, Youssef Elbanna, Thompson Trevor, Jesse Suarez

**Affiliations:** 1 Internal Medicine, HCA Florida Bayonet Point Hospital, Hudson, USA; 2 Endocrinology, Diabetes, and Metabolism, HCA Florida Bayonet Point Hospital, Hudson, USA

**Keywords:** chronic pancreatitis, c-peptide, insulin deficiency, pancreatogenic diabetes, partial pancreatectomy, secondary diabetes, severe hyperglycemia, type 3c diabetes mellitus

## Abstract

Pancreatogenic diabetes mellitus (type 3c diabetes mellitus, T3cDM) is an underrecognized form of secondary diabetes resulting from pancreatic exocrine disease, including chronic pancreatitis and pancreatic resection. It is frequently misclassified as type 2 diabetes mellitus, leading to delayed diagnosis and suboptimal management. We present a case of a 57-year-old female with a history of chronic alcoholic pancreatitis status post partial pancreatectomy who presented with severe uncontrolled hyperglycemia and medication nonadherence. Laboratory evaluation revealed a serum glucose of 668 mg/dL, hemoglobin A1c of 19%, and a low C-peptide level with normal insulin levels while receiving exogenous insulin, consistent with impaired endogenous insulin secretion and pancreatogenic diabetes. Despite repeated counseling and multiple attempts to optimize insulin therapy, the patient persistently refused insulin administration, resulting in refractory hyperglycemia throughout hospitalization. This case highlights the diagnostic challenges of distinguishing pancreatogenic diabetes from type 2 diabetes mellitus in patients with underlying pancreatic disease and emphasizes the importance of recognizing characteristic clinical features, including prior pancreatic injury and reduced endogenous insulin production. Early identification of T3cDM is essential because its pathophysiology and management differ substantially from type 2 diabetes mellitus and often require early insulin-based therapy to improve glycemic control and reduce long-term metabolic complications.

## Introduction

Pancreatogenic diabetes mellitus, also known as type 3c diabetes mellitus (T3cDM), is a form of secondary diabetes resulting from diseases affecting the exocrine pancreas, including chronic pancreatitis, pancreatic neoplasia, cystic fibrosis, and pancreatic resection [1,2]. Chronic pancreatitis is among the most common etiologies, with progressive pancreatic inflammation leading to irreversible destruction of both endocrine and exocrine tissue [3-5]. Despite increasing recognition, T3cDM remains underdiagnosed and is frequently misclassified as type 2 diabetes mellitus because of overlapping clinical features [2,6].

The American Diabetes Association (ADA) recommends screening for diabetes within three to six months following an episode of acute pancreatitis and annually thereafter, as well as annual screening for patients with chronic pancreatitis [1]. The pathophysiology of T3cDM differs substantially from that of type 2 diabetes mellitus and is characterized by impaired insulin secretion secondary to pancreatic β-cell loss, often accompanied by exocrine pancreatic insufficiency and nutritional deficiencies [2,6]. In contrast to patients with type 2 diabetes mellitus, those with T3cDM frequently demonstrate low or inappropriately normal C-peptide levels, reflecting reduced endogenous insulin production [2].

Management of pancreatogenic diabetes presents unique clinical challenges. Patients often experience brittle glycemic control and are at increased risk for both hyperglycemia and hypoglycemia because of the concomitant loss of glucagon-producing α-cells [6,7]. Current recommendations emphasize early initiation of insulin therapy in many patients, whereas incretin-based therapies are generally avoided because of concerns regarding pancreatitis risk [1,2].

We present a case of a patient with chronic alcoholic pancreatitis status post partial pancreatectomy who developed severe refractory hyperglycemia consistent with pancreatogenic diabetes mellitus. Beyond illustrating the importance of recognizing T3cDM in patients with structural pancreatic disease, this case highlights the diagnostic and therapeutic challenges posed by medication nonadherence, persistent insulin refusal, and psychosocial instability, emphasizing the need for individualized, multidisciplinary management.

## Case presentation

A 57-year-old female with a history of chronic alcoholic pancreatitis status post partial pancreatectomy, presumed type 2 diabetes mellitus, polysubstance use disorder, intravenous drug use, chronic spinal stenosis, and anxiety presented to the emergency department with severe uncontrolled hyperglycemia and worsening lower back pain following several recent falls. She also reported chills, polyuria, and shortness of breath but denied fever, nausea, vomiting, chest pain, abdominal pain, constipation, or diarrhea. The patient admitted that she was not taking any prescribed medications, including antihyperglycemic therapy, despite her history of diabetes mellitus.

Initial laboratory evaluation demonstrated severe hyperglycemia with a serum glucose level of 668 mg/dL (reference range = 74-106 mg/dL). Hemoglobin A1c was markedly elevated at 19%, consistent with longstanding poor glycemic control. Endocrine evaluation revealed a low C-peptide level of 0.87 ng/mL (reference range = 1.1-4.4 ng/mL), as summarized in Table 1. Serum insulin was within the reference range at 9.4 μIU/mL (reference range = 2.6-24.9 μIU/mL); however, the sample was obtained after initiation of exogenous insulin therapy and therefore did not reflect endogenous insulin secretion. The patient's history of chronic alcoholic pancreatitis, prior partial pancreatectomy, and low C-peptide level made pancreatogenic (type 3c) diabetes the most likely diagnosis.

**Table 1:** Initial laboratory findings. Initial laboratory findings demonstrating severe hyperglycemia and impaired endogenous insulin secretion. The combination of marked hyperglycemia (serum glucose = 668 mg/dL), elevated HbA1c (19.0%), low C-peptide (0.87 ng/mL), and the patient's history of chronic alcoholic pancreatitis with prior partial pancreatectomy strongly supported the diagnosis of pancreatogenic (type 3c) diabetes mellitus.

Laboratory test	Patient's result	Reference range	Clinical interpretation
Serum glucose	668 mg/dL	74-106 mg/dL	Severe hyperglycemia
Hemoglobin A1c	19.0%	<5.7%	Consistent with longstanding poor glycemic control
C-peptide	0.87 ng/mL	1.1-4.4 ng/mL	Decreased endogenous insulin production
Serum insulin	9.4 μIU/mL	2.6-24.9 μIU/mL	Within reference range; interpretation limited by exogenous insulin administration

During hospitalization, the patient underwent repeated counseling regarding the importance of glycemic control and insulin adherence. Multiple insulin regimens, including premixed insulin and basal-bolus therapy, were attempted; however, glycemic control remained poor because of repeated refusal of insulin administration.

## Discussion

T3cDM is an underrecognized form of secondary diabetes resulting from structural or functional pancreatic disease. Chronic pancreatitis is the leading cause of T3cDM, although pancreatic surgery, including partial pancreatectomy, also substantially increases the risk of endocrine insufficiency and diabetes development [3-6]. The prevalence of diabetes among patients with chronic pancreatitis increases with disease duration because of progressive destruction of pancreatic parenchyma [5,8]. Our patient had multiple features strongly suggestive of pancreatogenic diabetes, including chronic alcoholic pancreatitis, prior partial pancreatectomy, and severe hyperglycemia accompanied by a low C-peptide level, indicating markedly impaired endogenous insulin secretion rather than the insulin resistance that typically predominates in type 2 diabetes mellitus [2,6]. Although she had previously been diagnosed with type 2 diabetes mellitus, these clinical findings prompted reconsideration of the underlying etiology.

The diagnosis of T3cDM remains challenging because no universally accepted diagnostic criteria exist, and many patients are initially misclassified as having type 2 diabetes mellitus [6]. Ewald and Hardt proposed diagnostic criteria that include pancreatic exocrine insufficiency, pathological pancreatic imaging, and the absence of type 1 diabetes associated autoantibodies [4]. In our patient, pancreatic exocrine insufficiency was not formally assessed, available pancreatic imaging demonstrated postoperative changes consistent with prior partial pancreatectomy, and autoimmune diabetes associated antibodies, including glutamic acid decarboxylase 65 (GAD65), islet antigen-2 (IA-2), and zinc transporter 8 (ZnT8), were not obtained. These limitations precluded definitive confirmation using the proposed diagnostic criteria. Nevertheless, the combination of chronic alcoholic pancreatitis, prior pancreatic resection, low C-peptide level despite profound hyperglycemia, and the absence of clinical features strongly suggestive of autoimmune diabetes made pancreatogenic diabetes the most likely diagnosis.

Management of T3cDM differs substantially from that of type 2 diabetes mellitus because impaired insulin secretion is the predominant pathophysiologic mechanism. Insulin therapy is often required early in the disease course, particularly in patients with severe pancreatic damage or post-pancreatectomy diabetes [1,2]. Our patient's markedly elevated hemoglobin A1c and serum glucose reflected longstanding poor glycemic control and progressive β-cell dysfunction. Patients with T3cDM are generally considered less likely to develop diabetic ketoacidosis because pancreatic destruction results in the loss of both insulin-producing β-cells and glucagon-producing α-cells, thereby limiting ketogenesis despite severe insulin deficiency. However, diabetic ketoacidosis may still occur in the setting of profound insulin depletion or significant physiologic stress [9]. This pathophysiologic mechanism may explain why our patient did not develop diabetic ketoacidosis despite persistent severe hyperglycemia with serum glucose levels exceeding 400 mg/dL and a hemoglobin A1c of 19%.

Although the association between chronic pancreatitis, pancreatic resection, and T3cDM is well established [3-6], this case highlights the diagnostic challenges of distinguishing pancreatogenic diabetes from type 2 diabetes mellitus in patients with structural pancreatic disease. It underscores the importance of integrating pancreatic history, biochemical findings such as low C-peptide levels, and the overall clinical context to avoid diagnostic misclassification and guide appropriate management [2,4,6]. The case also illustrates the additional challenges posed by psychosocial instability and persistent insulin refusal despite repeated counseling, emphasizing that successful management of T3cDM requires not only appropriate insulin therapy but also individualized multidisciplinary strategies to address barriers to treatment adherence.

## Conclusions

Pancreatogenic diabetes mellitus is an underrecognized and frequently misclassified form of secondary diabetes that develops in the setting of chronic pancreatic disease and pancreatic resection. This case highlights the importance of maintaining a high index of suspicion for T3cDM in patients with a history of chronic pancreatitis or partial pancreatectomy who present with severe hyperglycemia and low C-peptide levels suggestive of impaired endogenous insulin secretion. Early recognition is essential because the pathophysiology and management of T3cDM differ substantially from those of type 2 diabetes mellitus and often require early insulin therapy. Greater awareness of T3cDM among clinicians may improve diagnostic accuracy, facilitate timely initiation of appropriate treatment, and ultimately reduce the risk of long-term metabolic complications.
